# Immune Therapies for Hematologic Malignancies

**DOI:** 10.3390/cancers13020295

**Published:** 2021-01-15

**Authors:** Matthew J. Olnes

**Affiliations:** 1 Hematology and Medical Oncology, Alaska Native Tribal Health Consortium, 3900 Ambassador Dr., Anchorage, AK 99508, USA; mjolnes@anthc.org; Tel.: +1-907-729-1180; Fax: +1-907-729-1189; 2 WWAMI School of Medical Education, University of Alaska Anchorage, 3211 Providence Drive, Anchorage, AK 99508, USA

The era of immunotherapy for hematologic malignancies began with the first allogeneic hematopoietic stem cell transplant (HSCT) study published by E. Donnall Thomas in 1957 [1]. Since then, the field of malignant hematology has been at the forefront of the clinical application of immune therapies. Allogeneic HSCT remains a standard treatment for acute leukemias and relapsed or refractory high-grade lymphomas, as well as select patients with relapsed and refractory indolent non-Hodgkin lymphomas and multiple myeloma. A key component of this therapy is the presence of allogeneic donor T-cells that provide long lasting immune surveillance and graft versus tumor effects. Some newer developments that have improved clinical outcomes include HSCT protocols that expand the pool of eligible donors with haploidentical grafts [2], and manipulations such as T-cell depletion to minimize rejection and graft versus host disease [3].

Allogeneic HSCT is the only curative treatment for myelodysplastic syndromes (MDS). Hypomethylating agents, lenolidomide, and luspatercept play an important role in managing this disease, but relapses invariably occur. Patients with low and intermediate risk MDS exhibit an increased oligoclonal expansion of autoreactive CD8+ T-cells which mount an immune assault on bone marrow progenitors, as well as reduced T helper 2 (T_H_2) and regulatory T-cell (T-reg) responses which favor autoimmunity [4,5]. Immune suppression administered to dampen the autoimmune attack on the bone marrow has a defined clinical role in a subset of patients with MDS [6], with some patients achieving long-term responses [7]. However, which MDS patients to treat with immune suppressive therapy remains a challenging question.

Chimeric antigen receptor (CAR) T-cells targeting CD19, CD20, and CD22 have become highly effective therapies for acute lymphoblastic leukemia (ALL) and for an increasing number of relapsed and refractory non-Hodgkin lymphoma subtypes, with many patients achieving durable long-term remissions [8,9,10,11]. There has been a recent surge in novel CAR T-cell constructs with a rapidly expanding variety of antigens being targeted, including CD30 to treat relapsed and refractory Hodgkin lymphoma [12], and CAR T-cells targeting B-cell membrane antigen (BCMA) and the G protein-coupled receptor class C group 5 member D (GPRC5D) to treat multiple myeloma [13,14,15]. Pre-clinical studies have also shown promise in targeting the interleukin-3 receptor alpha chain (IL-3Ra or CD123) [16] as well as CD13 and TIM3 to treat acute myeloid leukemia (AML) [17]. Mechanisms of CAR T-cell resistance have recently become further elucidated, including anti-mouse immune responses, sub-optimal CAR T-cell signaling, decreased persistence of CAR T-cells in vivo, ineffective tumor infiltration, and target antigen loss [14,18]. Strategies to overcome these resistance mechanisms are currently under investigation [14,18].

Therapeutic monoclonal antibodies (mAbs) targeting CD20 through antibody-dependent cytotoxicity have been in use to treat B-cell malignancies for more than two decades. CD38 and signaling lymphocytic activation molecule F7 have emerged as targets for mAbs to effectively treat relapsed and refractory multiple myeloma [19,20,21,22,23], and they are being investigated in the front-line setting [24]. Immune checkpoint inhibitor mAbs targeting programmed death ligand-1 (PD-1) have recently been approved as treatment options for relapsed and refractory Hodgkin lymphoma [25,26,27,28], and they are being investigated in other lymphoid malignancies [29]. There are also ongoing studies exploring the use of immune checkpoint inhibitors and the anti-CD47 directed monoclonal antibody (mAb) magrolimab in combination with hypomethylating agents to treat MDS [30,31].

Bispecific T-cell engaging mAbs (BiTEs) enhance cell-mediated immune responses by placing T-cells in close proximity to cells expressing target tumor antigens on their surface. Blinatumomab, a BiTE directed against CD19 and CD3, was approved as second line therapy for relapsed or refractory B-cell precursor ALL [32]. Blinatumomab combined with the tyrosine kinase inhibitor dasatinib recently demonstrated encouraging activity as a front-line chemotherapy-free therapy for Philadelphia chromosome positive ALL [33], and the BCMA and CD3 targeting BiTE AMG 420 exhibited activity in patients with relapsed and refractory multiple myeloma treated in a first-in-human clinical trial [34].

Antibody–drug conjugates (ADC) are another important immunotherapy for hematologic malignancies. ADCs are comprised of mAbs covalently linked to cytotoxic chemotherapy “warheads” [35]. Gemtuzumab ozogamicin is an ADC targeting CD33 that has an established role in treating patients with AML [36,37]. Brentuximab vedotin, an ADC directed against CD30, has become a treatment option for Hodgkin lymphoma [38,39] and peripheral T-cell lymphoma [40,41,42]. More recently, the ADCs directed against CD79b (polatuzumab vedotin) and CD19 (tafasitamab-cxix) were approved in the United States to treat patients with relapsed and refractory diffuse large B-cell lymphoma [43,44], and the BCMA directed ADC belantamab mafodotin-blmf was approved for patients with relapsed and refractory myeloma [45]. A plethora of other immunoconjugates are under investigation [35].

Natural Killer (NK) cells have evolved into a prominent immunotherapy for hematologic malignancies. Some attractive features of this approach are that NK cells function independently of major histocompatibility complex restriction, they do not require prior antigen sensitization, and they do not elicit graft versus host disease [46]. Sources for NK cell therapies include cells derived from haploidentical and umbilical cord donors, as well NK cell lines and memory-like cells induced by cytokines [47]. Strategies to activate and prolong NK cell function are an area of active clinical investigation [47,48,49]. A promising approach is the use of BiTEs and trispecific killer cell mAbs to enhance NK activity through the engagement of ligands such as CD16, IL-15R, NKG2D, and NKp46 [47,48,49,50]. CAR NK cell constructs have also shown clinical activity in treating CD19 positive lymphomas [51].

Many newer immune therapies share advantages inherent to allogeneic HSCT, including sustained immune surveillance and the propagation of anti-tumor killing. The recent exponential growth of immunotherapy studies indicates that the coming years will hold great promise for patients with hematologic malignancies as we move away from cytotoxic chemotherapy in favor of more effective strategies to engage the immune system therapeutically. This Special Issue of *Cancers* will highlight developments in pre-clinical research and the application of immune therapies for hematologic malignancies.
